# Cerebrospinal Fluid Cytokine Profiles Predict Risk of Early Mortality and Immune Reconstitution Inflammatory Syndrome in HIV-Associated Cryptococcal Meningitis

**DOI:** 10.1371/journal.ppat.1004754

**Published:** 2015-04-08

**Authors:** Joseph N. Jarvis, Graeme Meintjes, Tihana Bicanic, Viviana Buffa, Louise Hogan, Stephanie Mo, Gillian Tomlinson, Pascale Kropf, Mahdad Noursadeghi, Thomas S. Harrison

**Affiliations:** 1 Department of Clinical Research, Faculty of Infectious and Tropical Diseases, London School of Hygiene and Tropical Medicine, London, United Kingdom; 2 Botswana-UPenn Partnership, Gaborone, Botswana; 3 Division of Infectious Diseases, Department of Medicine, Perelman School of Medicine, University of Pennsylvania, Philadelphia, Pennsylvania, United States of America; 4 Desmond Tutu HIV Centre, Institute of Infectious Disease and Molecular Medicine, University of Cape Town, South Africa; 5 Institute of Infectious Disease and Molecular Medicine and Department of Medicine, University of Cape Town, South Africa; 6 Department of Medicine, Imperial College London, London, United Kingdom; 7 Research Centre for Infection and Immunity, Division of Clinical Sciences, St. George’s University of London, London, United Kingdom; 8 Division of Infection and Immunity, University College London, London, United Kingdom; Duke University, UNITED STATES

## Abstract

Understanding the host immune response during cryptococcal meningitis (CM) is of critical importance for the development of immunomodulatory therapies. We profiled the cerebrospinal fluid (CSF) immune-response in ninety patients with HIV-associated CM, and examined associations between immune phenotype and clinical outcome. CSF cytokine, chemokine, and macrophage activation marker concentrations were assayed at disease presentation, and associations between these parameters and microbiological and clinical outcomes were examined using principal component analysis (PCA). PCA demonstrated a co-correlated CSF cytokine and chemokine response consisting primarily of Th1, Th2, and Th17-type cytokines. The presence of this CSF cytokine response was associated with evidence of increased macrophage activation, more rapid clearance of *Cryptococci* from CSF, and survival at 2 weeks. The key components of this protective immune-response were interleukin (IL)-6 and interferon-γ, IL-4, IL-10 and IL-17 levels also made a modest positive contribution to the PC1 score. A second component of co-correlated chemokines was identified by PCA, consisting primarily of monocyte chemotactic protein-1 (MCP-1) and macrophage inflammatory protein-1α (MIP-1α). High CSF chemokine concentrations were associated with low peripheral CD4 cell counts and CSF lymphocyte counts and were predictive of immune reconstitution inflammatory syndrome (IRIS). In conclusion CSF cytokine and chemokine profiles predict risk of early mortality and IRIS in HIV-associated CM. We speculate that the presence of even minimal *Cryptococcus*-specific Th1-type CD4+ T-cell responses lead to increased recruitment of circulating lymphocytes and monocytes into the central nervous system (CNS), more effective activation of CNS macrophages and microglial cells, and faster organism clearance; while high CNS chemokine levels may predispose to over recruitment or inappropriate recruitment of immune cells to the CNS and IRIS following peripheral immune reconstitution with ART. These results provide a rational basis for future studies of immune modulation in CM, and demonstrate the potential of baseline immune profiling to identify CM patients most at risk of mortality and subsequent IRIS.

## Author Summary

Cryptococcal meningitis is a severe opportunistic infection, estimated to kill several hundred thousand HIV-infected individuals each year. One of the factors contributing to this high death toll is the inadequacy of antifungal treatments. As few novel antifungal drugs are being developed, several groups have started to investigate the potential of immune modulation, with treatments designed to change the patient’s immune response to infection. However, our understanding of the immune response to cryptococcal infection in HIV-infected patients, and how these responses impact on clinical outcomes, is limited. In this study, we took advantage of the fact that we can sample cerebrospinal fluid (CSF) from the site of the infection in patients when they develop cryptococcal meningitis. We undertook a detailed analysis measuring levels of immune response parameters in the CSF of these patients, and demonstrated that there were several distinct components of the immune response. Variations in these responses were associated with both the rate at which patients cleared their infection during treatment, and with mortality. Our results provide a basis for the development of future immunomodulatory therapies, and may allow identification of patients most at risk of dying, enabling more intensive treatments to be given to those at highest risk.

## Introduction

Cryptococcal meningitis (CM) is a leading cause of mortality in HIV-infected patients in low-resource settings [1, 2]. Current treatments for CM are inadequate, with high associated mortality [3–5], and high incidence of immune reconstitution inflammatory syndrome (IRIS) amongst those who survive and start antiretroviral therapy (ART) [6–8]. Understanding the host immune response to cryptococcal infection is important to enable rational development of immunomodulatory therapies, and also allow identification of patients most at risk of mortality and IRIS [3, 7, 9].

Much of our understanding of the immune response to *Cryptococcus* is derived from in-vitro and animal experiments. In these model systems effective immunity is dependent on robust CD4^+^ T-cell immune responses, the production of Th1-type cytokines such as interferon-γ (IFNγ), and “classical” activation of effector cells such as macrophages [10–17], leading to killing and clearance of infection. Th17-type CD4^+^ T-cell responses and cytokines appear to play a protective role [18–21], whilst Th2-type responses are associated with impaired control of infection and poor outcomes [16, 17, 22–26].

Human data are very limited. Epidemiological evidence clearly points to the importance of adequate CD4^+^ T-cell mediated immunity in the control of cryptococcal infection [27, 28], and experimental data suggest that the phenotype of the CD4^+^ T-cell response to *Cryptococcus neoformans* influences the outcome of CM [9, 29]. The importance of pro-inflammatory responses at the site of infection [7], and in particular IFNγ [30], for effective host immune responses to cryptococcal infection in HIV-infected patients has been reported. Preliminary data have not shown the Th1 / Th2 dichotomy seen in some mouse models, likely reflecting differences between carefully controlled animal models of cryptococcal infection and the complex situation in HIV-infected patients with heterogeneous immune status and organism burden [29], and no data are available on the role of Th17-type cytokines or the magnitude or phenotype of innate effector cell activation during infection.

To characterize the immune response to *Cryptococcus* during HIV-associated CM we measured cytokine concentrations, chemokine concentrations, and levels of macrophage activation markers in the cerebrospinal fluid (CSF) of ninety patients with CM enrolled in a clinical trial investigating the utility of short-course adjuvant IFNγ therapy [3]. The phenotype of the CSF immune response, and associations between the phenotype and disease burden at presentation, rate of clearance of infection, 2-week mortality, and development of IRIS were examined.

## Materials and Methods

### Participants and procedures

Ninety HIV-positive adults (age ≥ 21 years) with a first episode of cryptococcal meningitis, diagnosed by CSF India ink or cryptococcal antigen testing (titres ≥1:1024, Meridian Cryptococcal Latex Agglutination System; Meridian Bioscience Inc, Cincinnati, Ohio, USA), were enrolled sequentially between July 2007 and May 2010 into a clinical trial examining the effect of two different schedules of short-course adjuvant interferon-γ immunotherapy on the treatment of HIV-associated cryptococcal meningitis in Cape Town, South Africa [3]. The study has been described in detail elsewhere [3]. Detailed history and clinical examination findings were recorded at study enrolment. Lumbar punctures (LPs) with quantitative cerebrospinal fluid (CSF) cultures were performed on days 1, 3, 7 and 14. Cryptococcal clearance (early fungicidal activity, or EFA) was calculated as previously described [31]. All patients had CD4^+^-cell counts performed at study enrollment (FACSCount; Becton Dickinson). Patients were started on antiretroviral therapy consisting of stavudine/lamivudine/efavirenz at 2 to 4 weeks post commencement of antifungal therapy, and followed for one year. Mortality outcomes and the occurrence of IRIS (diagnosed according to a standardized definition [32]) were recorded.

### CSF cytokine analysis

CSF samples collected at day 1, 3, 7 and 14 were centrifuged, and the supernatant frozen at -80°C for subsequent quantification of cytokine concentrations. CSF IFNγ, tumor necrosis factor-α (TNFα), interleukin (IL)-2, IL-4, IL-6, IL-8 (chemokine (C-X-C motif) ligand 8), IL-10, IL-12p70, IL-17, IL-21, IL-22, IL-23, monocyte chemotactic protein-1 (MCP1, or chemokine (C-C motif) ligand 2), macrophage inflammatory protein-1α (MIP1α, or chemokine (C-C motif) ligand 3), RANTES (chemokine (C-C motif) ligand 5), granulocyte-macrophage colony-stimulating factor (GM-CSF, or colony stimulating factor 2), and vascular endothelial growth factor (VEGF) concentrations were measured in all patients using the Luminex multianalyte platform (Luminex) and Bio-Rad cytokine kits (Bio-Rad) [30]. The macrophage activation markers soluble CD14 (sCD14) and neopterin concentrations were measured in CSF using Bio-Rad and ELISA (ELItest Neopterin, BRAHMS Aktiengesellschaft, Hennigsdorf, Germany) kits respectively. The enzymatic activity of arginase and protein concentration of samples were measured as previously described [33]. This analysis focuses solely on the baseline assays performed prior to administration of antifungal therapy (results from subsequent time points are shown in S1 Fig All data are available as a supplementary S1 data file).

### Statistical analysis

Data were analysed using Stata version 13.0 (StataCorp, College Station, Texas, USA), R version 3.0.2 (R foundation for Statistical Computing), and Graphpad Prism version 6 (Graphpad Software Inc., San Diego, California, USA). Baseline CSF cytokine and chemokine concentrations were analysed using principal component analysis (PCA), a method of reducing complex correlated datasets into a series of linear, non-correlated “principal components” (PCs) and avoiding multiple comparisons. The first principal component accounts for as much of the variability in the data as possible, and each subsequent component accounts for the highest variance possible under the constraint that it is uncorrelated with preceding components [34, 35]. For PCA, analyte concentrations were log transformed, and normalized to the mean. Associations between the PCs and CD4^+^-cell count, CSF lymphocyte count, baseline fungal burden, rate of clearance of infection, and macrophage activation marker concentrations were examined using Pearson’s correlation coefficient. Associations between PCs and rate of clearance of infection (but not variables recorded at baseline) were adjusted for treatment group. Differences in PC scores between patients who survived and died, and those who did and did not develop IRIS, were examined using a linear regression model adjusting for treatment group, CD4^+^-cell count, and the previously described risk factors for mortality, baseline fungal burden and altered mental status [36]. A sensitivity analysis exploring differences in PC scores between patients who survived and died, and those who did and did not develop IRIS, was preformed limited to the patients in the trial control arm who did not receive adjuvant interferon-γ immunotherapy. For detailed analysis exploring the relationships between individual cytokines, associations between continuous variables were explored using Pearson’s correlation coefficient and linear regression analysis. Variables were compared across groups using Student’s t-tests, Kruskal-Wallis tests, χ^2^ tests, or Fisher’s Exact tests as appropriate. Comparisons of paired groups were made using the Wilcoxon matched pairs test. Permutation tests, with 5000 permutations, were used to control the family wise-error rate (FWER) for the fifteen correlations / partial correlations between the cytokines IL-4, IL-10, IL-17, MCP-1 and baseline and outcome variables. Permutation tests used the max statistic for adjusting the p-values to ensure FWER of 0.05 while allowing for the potentially strong correlations between variables. Unadjusted p-values for those associations that maintained a FWER<0.05 across the multiple comparisons are reported. Support vector machine (SVM) learning and decision tree analyses were used to assess the capacity for baseline CSF cytokine measurements to predict mortality or IRIS. The support vector machines algorithm was implemented in R v2.15.1 using the kernlab package and ksvm function with a linear kernel (vanilladot), cost 1 and leave one out cross validation. Decision tree classification was performed using Waikato Environment for Knowledge Analysis (WEKA) v 3.6.9 using different decision tree classifiers (Alternating Decisions Tree, Simple Cart, J48 Decision Tree and Random Forrest) with leave one out cross validation. For the purposes of all analyses the two IFNγ treatment groups were considered as a single “IFNγ treated” group. Statistical significance was defined as p≤0.05.

### Ethics statement

The study was approved by the Human Research Ethics Committee of the University of Cape Town and St. George’s University of London. Patients gave written informed consent for blood and CSF samples to be used for research purposes.

## Results

CSF samples were available from all 90 patients enrolled in the clinical trial. The median age was 32 years (IQR 28–38), 44% (40) were male, and the median CD4^+^ cell count was 27 cells/μL (IQR 14–51). All patients were treated with amphotericin B 1mg/kg plus flucytosine 100mg/kg daily for fourteen days. Twenty nine patients received adjunctive IFNγ 100μg subcutaneously on days 1 and 3, and thirty patients received IFNγ 100μg subcutaneously thrice weekly for the first two weeks of therapy. Mortality at 2-weeks was 16% (14). All patients were ART naïve at study enrollment, and ART was initiated at a median of 23 days post initiation of antifungal therapy. IRIS occurred in 14% of those who started ART (9/65), occurring a median of 60 days post ART initiation (Table 1).

**Table 1:** Baseline characteristics of the patients.

Variable	All patients (n = 90)
Age (years)	32 (28–38)
Sex (% male)	44% (40)
Abnormal mental status (%)	37% (33)
Baseline CD4 cell count (x 10^6^/ml)	27 (14–51)
HIV viral load (copies/ml)	120,000 (28,000–300,000)
CSF lymphocyte count (x 10^6^/L)	14 (0–72)
CSF protein (g/dL)	0.97 (0.55–1.54)
India ink +ve (%)	92% (83)
Baseline fungal burden (CFU/ml CSF)	240,000 (36,500–745,000)
2 week mortality (%)*	16% (14)
IRIS (%)* ^†^	14% (9)

Results are median inter-quartile range (IQR) unless indicated. CFU, colony forming unit; CSF, cerebrospinal fluid; IRIS, immune reconstitution inflammatory syndrome.

*All patients were treated with Amphotericin B 1 mg/kg plus flucytosine 100 mg/kg per day for 14 days (“standard treatment”). 31 patients received standard treatment alone (“controls”). 29 patients received standard treatment plus IFNγ 2 doses (100 μg days 1 and 3). 30 patients received standard treatment plus IFNγ 6 doses (IFNγ 100 μg days 1,3,5,8,10, and 12). There were no significant differences in either mortality or frequency of IRIS between treatment groups [3].

^†^IRIS is expressed as percentage of patients who initiated antiretroviral therapy (n = 65)

### CSF cytokine and chemokine responses

**Fig 1:**
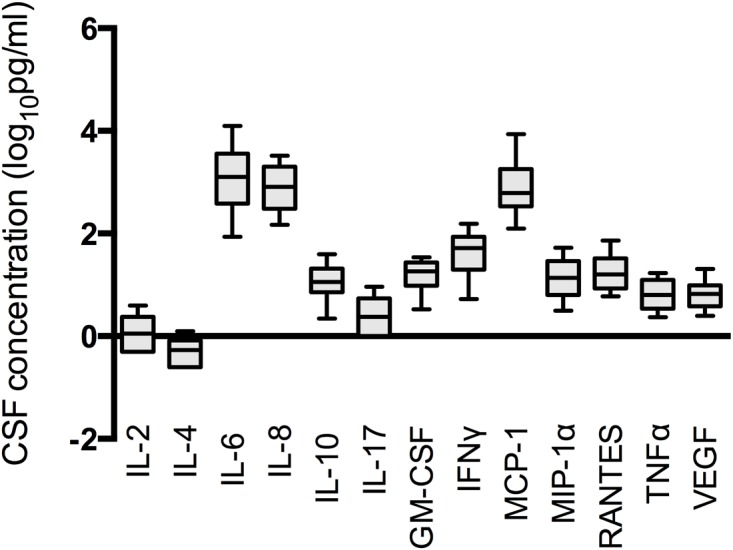
Baseline cerebrospinal fluid cytokine and chemokine concentrations in a cohort of ninety patients with HIV-associated cryptococcal meningitis. Baseline concentrations of the thirteen cytokines and chemokines measured and detected in the CSF of patients with HIV-associated cryptococcal meningitis are shown. Cytokine and chemokine concentrations were log transformed to approximate normal distributions. Filled bars are the inter-quartile range with a line at the median, with error bars to the 10^th^ and 90^th^ centiles. IL-12, IL-21, IL-22 and IL-23 concentrations were below the limit of detection in the majority of cases.

**Fig 2:**
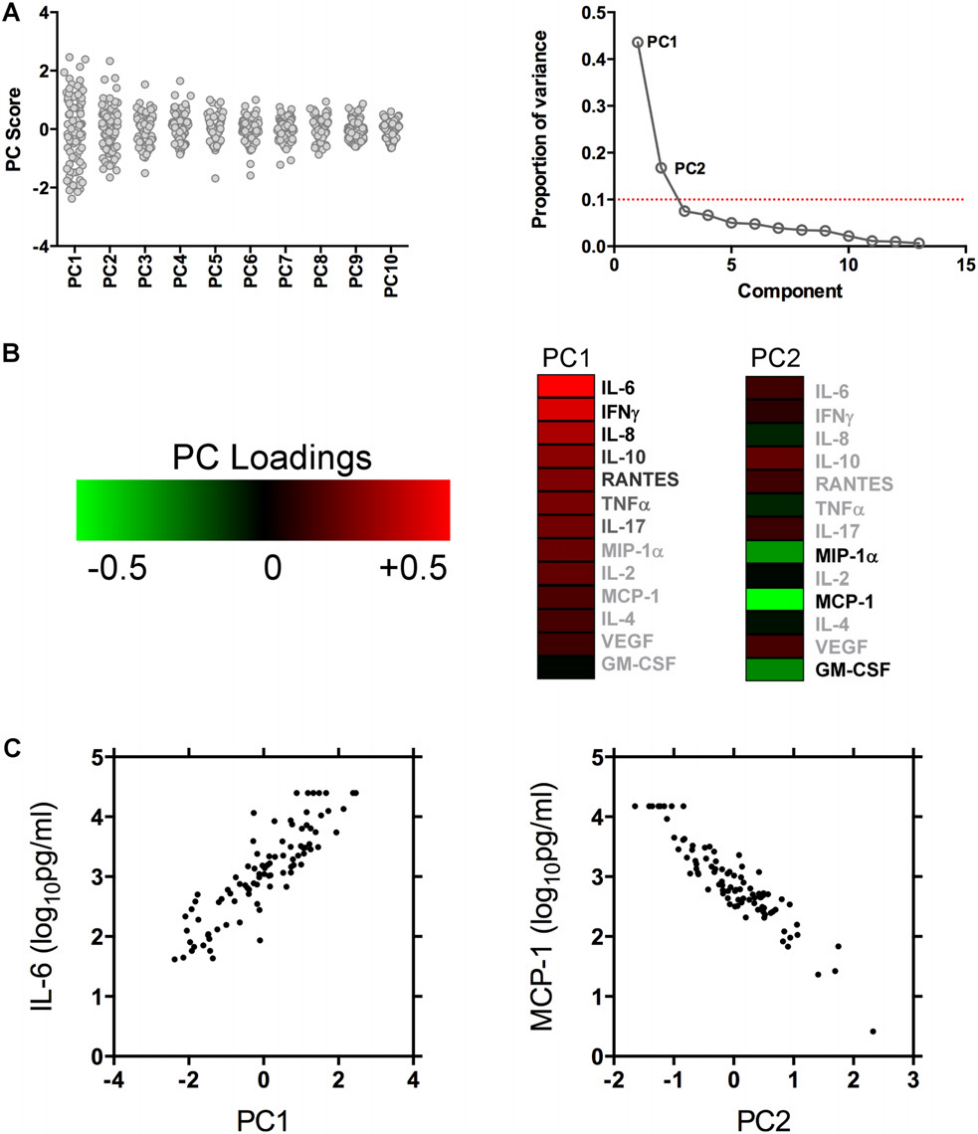
Principal component analysis scores and weightings. Figure (A) shows the variance of each of the principal component scores amongst the 90 patients studied. Figure (B) shows the proportion of variance in the dataset explained by each of the principal components. The majority of the variance in the dataset was explained by the first two principal components, which were used to explore associations between CNS immune responses and outcome. Contribution of each variable to the variance between patients in PC1 and PC2 is shown in the heat map. PC1 was composed primarily of pro-inflammatory cytokines IL-6, IFNγ and the chemokine IL-8. PC2 contained a heavy negative weighting of chemokines MCP-1, MIP-1α and the cytokine GM-CSF. Figure (C) shows the associations between PC1 and baseline CSF IL-6 concentrations, and PC2 and baseline CSF MCP-1 concentrations. See S2 Fig for more detailed PC1 and PC2 associations.

**Fig 3:**
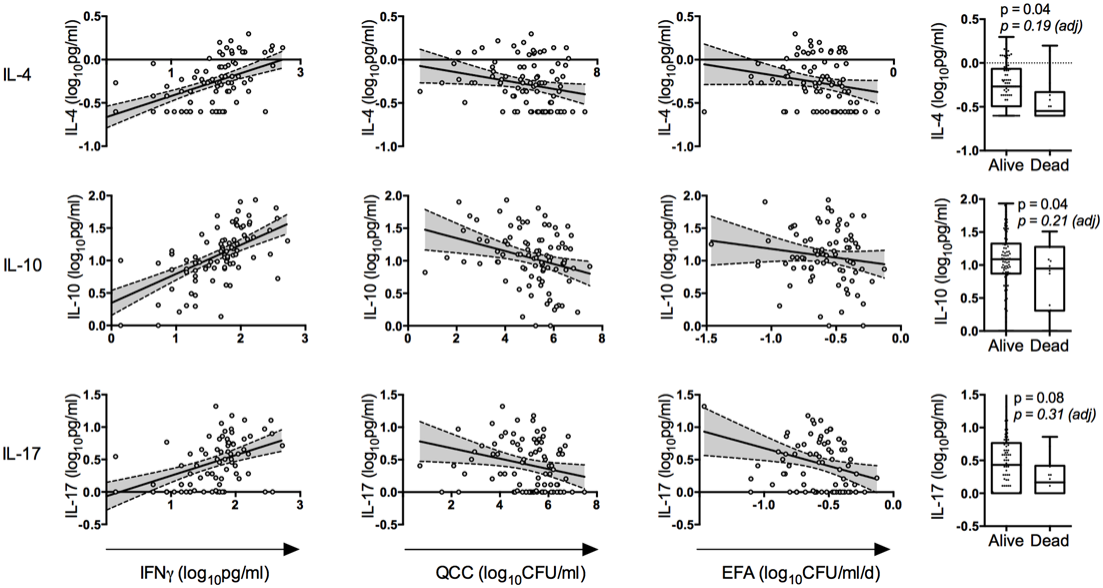
Relationships between cerebrospinal fluid interferon-γ, IL-4, IL-10 and IL-17 concentrations, and their associations with disease severity and outcome. Associations between baseline IL-4 (top row), IL-10 (middle row) and IL-17 concentrations (bottom row), and baseline IFNγ concentrations, baseline fungal burden (quantitative cryptococcal culture, QCC), rate of clearance of infection (early fungicidal activity, EFA) and 2 week mortality. Best-fit regression lines are shown with 95% confidence intervals. Box plots show the median and extend to the inter-quartile range, with whiskers denoting minimum and maximum values. P-values derived from Student’s t-tests are shown for the mortality associations (both unadjusted and adjusted for family wise error rate using permutation testing). All EFA and mortality associations were adjusted for treatment group. Pearson’s correlation coefficients and significance levels were: IL-4/IFNγ r = 0.57 p<0.001, IL-4/QCC r = -0.23 p = 0.03, IL-4/EFA r = -0.21 p = 0.1. IL-10/IFNγ r = 0.64 p<0.001, IL-10/QCC r = -0.30 p = 0.005, IL-10/EFA r = -0.15 p = 0.2. IL-17/IFNγ r = 0.47 p<0.001, IL-17/QCC r = -0.25 p = 0.02, IL-17/EFA r = -0.29 p = 0.03. These associations all remained significant when controlling for a family wise error rate of 0.05.

Baseline CSF cytokine concentrations are shown in Fig 1. IL-12, IL-21, IL-22 and IL-23 concentrations were below the limit of detection in the majority of cases, and have been excluded from subsequent analyses. Principal component analysis was used to identify co-correlated cytokine and chemokine measurements that accounted for the variance across the data set. The majority of the variance was reflected by PC1 (44%) and PC2 (17%) (Fig 2A). The component loadings for each variable showed that the variance in PC1 was driven by positive loading scores for the pro-inflammatory cytokines IL-6, IFNγ and the chemokine IL-8 (Fig 2B). IL4, IL10 and IL17 levels also made positive, albeit more modest contribution to the PC1 score, suggesting that Th1 (IFNγ), Th2 (IL4 and IL10) and Th17 (IL17) responses were co-correlated in this context and confirmed by direct pairwise comparisons of each cytokine (Fig 3). The variance in PC2 was due to negative loading scores for the chemokines MCP-1, MIP-1α, and the cytokine GM-CSF (Fig 2B). Hence PC1scores were positively correlated with the levels of the pro-inflammatory cytokines IL-6 and IFNγ and PC2 scores were inversely correlated with the levels of the chemokines MCP-1 and MIP-1α (Fig 2C and S2 Fig).

### CSF cytokine and chemokine correlations with microbiological, immunological, and clinical variables and outcomes in cryptococcal meningitis

PCA indicated that co-correlated pro-inflammatory cytokines and chemokines represented by PC1 scores, and those represented by PC2 scores function as independent variables. To assess whether proinflammatory cytokines or chemokines were related to other clinically important variables in cryptococcal meningitis we explored the relationship of PC1 and PC2 with peripheral blood CD4, CSF lymphocyte counts, markers of CSF macrophage (or resident CNS macrophage like cell) activation, CSF arginase activity, baseline CSF fungal burden, rate of clearance of infection, 2-week mortality and IRIS (Table 2 and Table 3). PC1 scores showed a weak positive relationship with CD4 count and stronger relationship with CSF lymphocytes. PC2 scores correlated with both of these variables. These suggest that low levels of both peripheral CD4 cell count and CSF lymphocyte count were associated with reduced levels of pro-inflammatory cytokines but increased levels of the chemokines MCP-1 and MIP-1α in the CSF. Both PC1 and PC2 were also strongly positively correlated with the two soluble markers of macrophage activation in CSF, neopterin and sCD14, however not with arginase activity (Table 2), indicating that macrophage activation was associated with robust pro-inflammatory CSF cytokine responses, and reduced chemokine expression. There were no significant associations between arginase activity and either IFNγ, IL-10, or IL-4.

**Table 2:** Correlations between principal component (PC) scores and baseline microbiological, immunological and clinical variables.

	PC1		PC2	
Variable	Pearson’s-r	*p-value*	Pearson’s-r	*p-value*
Baseline CD4 cell count (x10^6^/ml)	0.26	*0*.*02*	0.34	*0*.*001*
CSF lymphocyte count (x10^6^/L)	0.40	*<0*.*001*	0.57	*<0*.*001*
CSF Neopterin (log_10_pg/ml)	0.55	*<0*.*001*	0.30	*0*.*004*
CSF sCD14 (log_10_pg/ml)	0.52	*<0*.*001*	0.26	*0*.*01*
CSF arginase (units)	0.02	*0*.*9*		
Baseline fungal burden (CFU/ml CSF)	-0.23	*0*.*03*	-0.51	<0.001
Early fungicidal activity (log_10_CFU/ml/day)*	-0.28	*0*.*03*	-0.21	0.04

*The association between PC1 score and EFA remained significant in a linear regression model adjusted for treatment group (p = 0.03). Unadjusted p-value = 0.01.

**Table 3:** Associations between principal component (PC) scores and outcome.

		PC1			PC2		
		Mean (SE)	*Delta* ^†^	*p-value*	Mean (SE)	*Delta* ^†^	*p-value*
Mortality (2 weeks)^†^	Alive	0.09 (0.12)	*-0*.*95*	*0*.*01*	0.01 (0.09)	*-0*.*05*	*0*.*8*
	Dead	-0.49 (0.44)			-0.08 (0.13)		
IRIS^¶^	No IRIS	0.14 (0.12)	*-0*.*08*	*0*.*3*	0.12 (0.09)	*-0*.*63*	*0*.*004*
	IRIS	-0.25 (0.48)			-0.75 (0.18)		

^†^The adjusted p values shown were derived from a linear regression model adjusting for treatment group, CD4^+^-cell count, and the previously described risk factors for mortality, baseline fungal burden and altered mental status. The adjusted delta is the β-coefficient derived from the adjusted linear regression model. Unadjusted p-values were 0.09 (PC1) and 0.7 (PC2).

^¶^The association between PC2 and IRIS remained significant in both the linear regression model adjusting for treatment group, CD4^+^-cell count, and the previously described risk factors for mortality, baseline fungal burden and altered mental status, as described above (*p = 0*.*004*), and a linear regression model (results shown in table) adjusting for treatment group and the known risk factors for IRIS, baseline fungal burden and CSF white cell count *(p = 0*.*004*). Unadjusted p-value = 0.001. The adjusted delta is the β-coefficient derived from the adjusted linear regression model.

**Fig 4:**
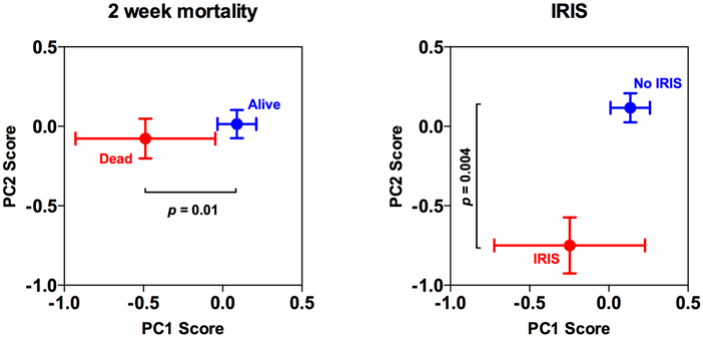
Associations between baseline cerebrospinal fluid immune response profiles and clinical outcome. Associations between baseline PC1 and PC2 scores and 2-week mortality and IRIS. The points represent the mean values, with standard errors denoted by the error bars. The adjusted p value are shown, derived from (in the case of mortality) a linear regression model adjusting for treatment group, CD4^+^-cell count, and the previously described risk factors for mortality, baseline fungal burden and altered mental status; and (in the case of IRIS) a linear regression model adjusting for treatment group, CD4^+^-cell count, and the previously described risk factors for IRIS, baseline fungal burden and CSF white cell count.

Fungal burden was negatively correlated with PC1 and PC2 indicating that patients with high fungal burdens tended to have pauci-inflammatory CSF cytokine profiles with high MCP-1 and MIP-1α chemokine expression, while lower fungal burdens were associated with more robust CSF inflammatory responses and lower chemokine levels. Importantly PC1 scores showed a positive correlation with more rapid clearance of cryptococci from the CSF (an association that remained significant after adjustment for treatment group), consistent with the hypothesis that robust pro-inflammatory responses contribute to fungal clearance. Also in keeping with this observation, PC1 scores were lower in patients who had died within 2 weeks compared to those who survived (Fig 4). This association was significant following adjustment for treatment group, CD4 count, and the key predictors of mortality, baseline fungal burden and altered mental status (p = 0.01). There were no significant associations between PC2 and mortality (Fig 4).

Amongst patients who survived, PC2 but not PC1 scores, differentiated patients who developed IRIS following the initiation of ART (Fig 4). PC2 scores in patients who developed IRIS were significantly lower than those who did not, showing that high chemokine expression at baseline, associated with low peripheral CD4 counts and CSF lymphocyte counts, was predictive of subsequent IRIS (Fig 4). The association between PC2 score and IRIS remained significant in analyses adjusted for treatment group, CD4^+^-cell count, and the previously described risk factors for mortality, baseline fungal burden and altered mental status, as described above (p = 0.004), and in a linear regression model adjusting for treatment group and the known risk factors for IRIS, baseline fungal burden and CSF white cell count (p = 0.004).

**Fig 5:**
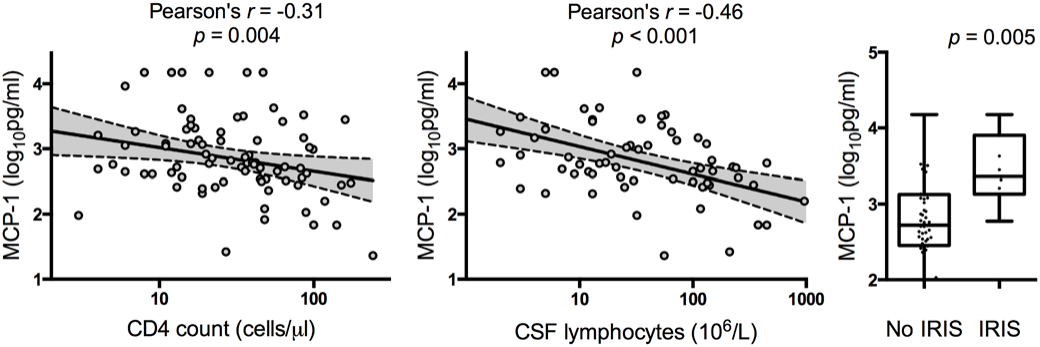
Relationship between CSF MCP-1 concentrations and CD4 cell counts, cerebrospinal fluid lymphocyte counts, and immune reconstitution syndrome. Associations between baseline MCP-1 concentrations and baseline CD4 cell count, concentrations, cerebrospinal fluid (CSF) lymphocyte count and immune reconstitution syndrome (IRIS). The IRIS association was adjusted for treatment group. Best-fit regression lines are shown with 95% confidence intervals. Box plots show the median and extend to the inter-quartile range, with whiskers denoting minimum and maximum values. These associations all remained significant when controlling for a family wise error rate of 0.05.There was no significant difference in CSF MCP-1 concentrations between those who survived (749.1 pg/ml) and those who died (858.4 pg/ml), *p = 0*.*76*.

The largest contributor to PC2 was MCP-1, which was individually strongly negatively correlated with CD4 count (r = -0.31, p = 0.004) and log_10_ CSF lymphocyte count (r = -0.46, p<0.001), and associated with IRIS (baseline geometric mean CSF MCP-1 concentration 574.7 pg/ml in those not developing IRIS versus 2887.0 pg/ml in those who subsequently developed IRIS, p = 0.005, Fig 5). These associations all remained significant when controlling for a FWER of 0.05.

A sensitivity analysis examining associations between PC scores and outcomes restricted to the patients in the trial control arm who did not receive adjuvant interferon-γ immunotherapy was performed. In this smaller sample, the association between PC1 and rate of clearance of infection from the CSF and mortality were no longer significant (early fungicidal activity Pearson’s-r = -0.12, p = 0.5; mortality PC1 delta = -0.01, p = 0.9). The associations between PC2 and IRIS remained significant in both crude (p = 0.01) and adjusted analysis (p = 0.03).

### Stratification of clinical outcomes by baseline CSF cytokine and chemokine measurements

Taken together, our data suggest that multiparameter measurements of CSF cytokines and chemokines in patients presenting with cryptococcal meningitis may predict the risk of early mortality or development of IRIS following ART. To test whether our data could be used to predict these clinical outcomes, we employed machine-learning algorithms and leave one out cross-validation to classify each patient after training on the data from all the other patients in the study. To take advantage of high dimensional pattern recognition we used support vector machine learning; and to assess whether a minimum number of variables could classify patients correctly, decision tree analyses was used (Table 4). Support vector machine learning (SVM) correctly predicted mortality or survival at two weeks in 84% of patients and IRIS in 83% of patients. Using a variety of decision tree analyses, we were also able to predict mortality or survival at two weeks with 80-84% accuracy and IRIS with 78-84% accuracy.

**Table 4:** 

Classification tool		Cross validation error (%)	
		Mortality	IRIS
Support vector machine learning		15.56	16.9
Decision Tree analyses	Alternating decision tree	20	18.5
	J48 Algorithm	15.6	21.5
	Simple Cart	16.7	15.4
	Random Forrest	20	16.9

## Discussion

The phenotype of the baseline CSF immune response in patients with HIV-associated CM was shown to be associated with both microbiological and clinical outcomes, and predictive of subsequent IRIS. For the first time we have demonstrated that more robust pro-inflammatory CNS cytokine responses, characterized by higher levels of CSF IL-6 and IFNγ are associated with evidence of increased markers of innate effector cell activation, and, in keeping with previous reports [30], more effective control of fungal burden, faster clearance of infection during treatment, and survival. These findings are consistent with animal model data demonstrating the importance of pro-inflammatory CNS cytokine responses [13, 37, 38] and microglial cell activation [38, 39] in response to cytokines such as IFNγ for host resistance to cryptococcal infection. They also support previous findings showing that higher expression of pro-inflammatory cytokines such as IFNγ and TNFα by peripheral CD4^+^ T-cells [29], and higher levels of IFNγ, TNFα, IL-6, and IL-8 in the CSF of patients with HIV-associated CM [30] are associated with improved outcomes.

In keeping with animal models [12, 13, 19, 23, 40], our data suggests that the Th-17-type cytokine, IL-17, plays an important role in the human CNS immune response to *Cryptococcus*. IL-17 concentrations were closely correlated with IFNγ levels, and associated with rate of clearance of infection and survival. Of note, IL-17 production was not observed in response to cryptococcal mannoprotein stimulation of CD4^+^ T-cells from a subset of patients included in this study[29], suggesting that the source of CSF IL-17 may not be Th17-type CD4^+^ T-cells. Other cytokines associated with Th17-type T-cell responses, including IL-21, IL-22 and IL-23 were not present in detectable quantities in the majority of patients studied, further supporting this supposition. γδ^+^ T-cells, CD8^+^ T-cells, NK cells, and neutrophils are all capable of producing the cytokine [21, 40–44], although it is not possible to determine the source of CSF IL-17 from our data.

We found no evidence for a detrimental Th2-type response in CM patients, nor evidence for arginase activity, which in mouse models of *Cryptococcal* infection is associated with alternative activation of effector cells [16]. Levels of the classic Th2 cytokine IL-4 correlated closely with IFNγ concentrations, as did IL-10 concentrations, and both were associated with better control and clearance of cryptococcal infection and lower mortality (although the mortality association with these individual cytokines did not remain statistically significant following adjustment for multiple comparisons, requiring validation in future studies). Th2-type T-cell responses, characterized by production of IL-4 and IL-13 and alternative activation of macrophages, are strongly associated with adverse outcomes in mouse models of cryptococcal infection [45]. Reasons for the differences between our findings and the animal models could relate to intrinsic differences between mouse and human immune systems, particularly in the context of advanced HIV-infection. The Th1/Th2 dichotomy described in mouse models appears less pronounced in humans [46], and arginase production, which has been shown to be a marker of disease severity in HIV-infected patients [47], is not well described in human macrophages, but rather is associated with myeloid derived suppressor cells and neutrophils [47]. It also almost certainly reflects the fact that the patients in our study had been infected with *Cryptococcus* for some time, and had had active CNS disease for several weeks prior to presentation [3]. Many of the mouse studies have examined local pulmonary responses during early infection, prior to dissemination of infection, and not CSF cytokine profiles. Additionally mouse models do not incorporate HIV-infection, or the effects of antifungal treatment. It is possible that a Th1/Th2 dichotomy exists during early infection in humans, but is not reflected in the CSF during the later stages of disease. Recent evidence suggests that both T-cell and macrophage polarization are dynamic and plastic processes [46, 48], and the CSF immune responses observed in the study patients likely represent a complex interaction of pro-inflammatory, compensatory anti-inflammatory and tissue repair processes that develop as disease evolves.

In terms of cryptococcal IRIS, previous studies have identified poor baseline inflammatory responses, rapid immune reconstitution from this low baseline, and a high organism or antigen burdens as key risk factors [6]. Low peripheral CD4 counts and CSF lymphocyte counts, and reduced levels of IFNγ, TNFα, IL-6, and IL-8 in the CSF at disease presentation have all been associated with increased risk of subsequent IRIS [7–9, 49, 50]. Although, in our study, patients who developed IRIS did tend to have a less inflammatory CSF cytokine response at baseline, the more striking finding was that development of cryptococcal IRIS following ART initiation was strongly associated with high CNS expression of the chemokines MCP-1, MIP-1α, and the cytokine GM-CSF at initial CM presentation. This finding provides support for the findings of Chang et al., who recently demonstrated that increased CNS expression of MCP-1 and MIP1α at baseline were associated with subsequent IRIS, and hypothesized that CD8+ T-cell and myeloid cell trafficking into the CNS in response to these chemokines predisposed to aberrant immune responses and excessive CNS inflammation following ART initiation [49].

In a previous study we have demonstrated that the phenotype of the peripheral CD4^+^ T-cell response to *Cryptococcus* is associated with disease severity and outcome in HIV-associated CM [29]. IFNγ and TNFα predominant responses were associated with increased CSF lymphocyte counts, higher levels of CSF cytokines including IL-17, and survival. This study builds on these prior findings, suggesting that the presence of relatively small numbers of IFNγ and TNFα-producing *Cryptococcus*-specific memory CD4^+^ T-cells are capable of promoting a cellular infiltrate into the CNS in response to local production of chemokines such as MCP-1 and MIP1α by microglial cells following exposure to *Cryptococci*. We hypothesize that these infiltrating cells then stimulate expression of pro-inflammatory cytokines by both resident glial cells and trafficked cells of myeloid and lymphoid lineages, leading to activation of microglial cells and macrophages, with resultant restriction of intracellular growth of *Cryptococci*, resulting in lower fungal burdens at presentation, faster clearance on infection on treatment, and improved survival. With the evolution of this protective, predominantly Th1-type inflammatory cytokine response, compensatory parallel increases in both Th2-type cytokines such as IL-4 and regulatory cytokines such as IL-10 occur. In the absence of any effective *Cryptococcus*-specific CD4 response, microglial cells and CNS macrophages are unable to effectively restrict cryptococcal growth, leading to high organism burdens, poor clearance of infection during treatment, and high mortality. These patients with severe immune suppression at baseline, indicated by low peripheral CD4 counts and CSF lymphocyte counts, also have markedly up regulated CNS MCP-1 and MIP1α chemokine expression, which, following immune restoration with ART, leads to an influx of inflammatory cells, excessive local inflammation, and IRIS.

Despite the prospective nature of our study, with comprehensive clinical and immunological data, there are a number of limitations. Firstly, the cellular source of the CNS cytokine production is not possible to elucidate from these data. Unlike in previous smaller studies [30, 51], levels of CSF cytokines were significantly correlated with both peripheral CD4^+^ cell count and CSF lymphocyte count. This may suggest that the source of these CSF cytokines is infiltrating T-cells and monocytes (which may be mistaken for lymphocytes on CSF cell counts). Alternatively such incoming cells may facilitate or stimulate cytokine production by resident CNS immune cells. Interestingly a consistent and significant inverse relationship was seen between the levels of the chemo-attractant chemokines MCP-1, MIP-1α and peripheral CD4^+^ cell count and CSF lymphocyte count. This finding is consistent with existing data suggesting that chemokine production by glial cells and monocytes/macrophages is not significantly compromised in patients with late stage HIV-infection [52], and that the poor CNS inflammatory response observed is primarily the result of the absence of effective T-cell support. A biological feedback loop appears to be in operation such that chemokine production is rapidly down-regulated in the face of even minimal local lymphocyte infiltration and CD4^+^ T-cell help. Secondly, when exploring the correlations between baseline CSF immune parameters and fungal burdens it is not possible to definitively attribute causality. Whilst it seems plausible that the association between more vigorous CSF inflammatory responses and lower organism burdens means that variation in the patient’s ability to mount an effective inflammatory response plays a key role in determining disease severity, it is possible that very high organism burdens paradoxically lead to down regulation of the host inflammatory responses. Finally, these data were derived from a clinical trial comparing differing three treatment regimens leading to different rates of fungal clearance (but not significant differences in mortality or rates of IRIS). The cytokine and chemokine levels used in these analyses were all determined prior to the administration of any antifungal therapy, meaning all baseline comparisons are unaffected by treatment group. And in all analyses relating to rate of clearance of infection, IRIS, and mortality outcomes, adjustment for treatment group was made. In each of these cases the associations seen in unadjusted analysis remained robust, or were strengthened following adjustment, supporting the validity of our findings. The sensitivity analysis restricted to the study control group (i.e. those who did not receive adjuvant interferon-γ immunotherapy) was underpowered as a result of the small sample size. However even in this small sample, although the mortality associations were no longer significant, the association between PC2 and IRIS remained statistically robust.

In conclusion, the presence of a CSF inflammatory response consisting of Th1, Th2 and Th17 type cytokines has been shown to correlate with markers of innate effector cell activation, more rapid clearance of infection and survival in patients with HIV-associated cryptococcal meningitis. We did not find evidence of a dichotomous Th1/Th2 response. Rather Th1, Th2 and Th17 type cytokine concentrations correlate closely with each other, and the presence of this combined inflammatory response is beneficial, while its absence is detrimental. Patients with low peripheral CD4^+^ T-cell counts and CSF lymphocyte counts at disease presentation had high CNS chemokine expression, and were at high risk of subsequent IRIS following ART initiation.

## Supporting Information

S1 Fig The time course of the CSF immune response. Median chemokine and cytokine levels, with error bars to 75^th^ centile, are shown on days 1, 3, 7, and 14. No significant differences in the change in cytokine levels between days 1 and 3 were seen in the interferon-γ treated patients compared to controls. Between day 1 and day 7 there were larger reductions in IL-6, IL-17, RANTES and VEGF concentrations in IFNγ treated patients than controls, mirroring the more rapid decline in fungal burden (IL-6 5861pg/ml reduction versus 1500pg/ml, p = 0.03; IL-17 6.3pg/ml reduction versus 2.7pg/ml, p = 0.007; RANTES 16.7pg/ml versus 11.8pg/ml, p = 0.05; and VEGF 1.1pg/ml reduction versus 0.8pg/ml increase, p = 0.03).
(TIFF)

S2 Fig Principal component correlations. The associations between PC1 and baseline CSF IL-6, IFNγ, and IL-8 concentrations; and PC2 and baseline CSF MCP-1, MIP-1α, and GM-CSF concentrations.
(TIFF)

S1 Data File A stata dataset containing raw anonymized baseline clinical data and log transformed cytokine, chemokine, and macrophage activation marker levels from study days 1, 3, 7, and 14 from the 90 study patients. Variables included are adjuvant treatment category (interferon gamma administered), CD4 cell count (cells/μL), abnormal mental-status (defined as a Glasgow Coma Score <15 or seizures), CSF lymphocyte count (x 10^6^/L), baseline fungal burden (quantitative cryptococcal culture, log_10_CFU/ml), rate of clearance of infection (early fungicidal activity, log_10_CFU/ml/day), day 1 CSF opening pressure (cm H_2_O), mortality at 2 weeks, antiretroviral initiation status, immune reconstitution inflammatory syndrome (IRIS), and day 1, 3, 7, and 14 CSF levels of interferon-γ (IFNγ), tumor necrosis factor-α (TNFα), interleukin (IL)-2, IL-4, IL-6, IL-8 (chemokine (C-X-C motif) ligand 8), IL-10, IL-12p70, IL-17, IL-21, IL-22, IL-23, monocyte chemotactic protein-1 (MCP1, or chemokine (C-C motif) ligand 2), macrophage inflammatory protein-1α (MIP1α, or chemokine (C-C motif) ligand 3), RANTES (chemokine (C-C motif) ligand 5), granulocyte-macrophage colony-stimulating factor (GM-CSF, or colony stimulating factor 2), vascular endothelial growth factor (VEGF), soluble CD14 (sCD14) and neopterin concentrations (log_10_picograms/mL), and enzymatic activity of arginase.
(DTA)
