# Supplementary figures and images for: Cerebrospinal Fluid Cytokine Profiles Predict Risk of Early Mortality and Immune Reconstitution Inflammatory Syndrome in HIV-Associated Cryptococcal Meningitis
Source: PLoS Pathog. 2015 Apr 8;11(4):e1004754. doi: 10.1371/journal.ppat.1004754 (PMC4390200; doi:10.1371/journal.ppat.1004754)

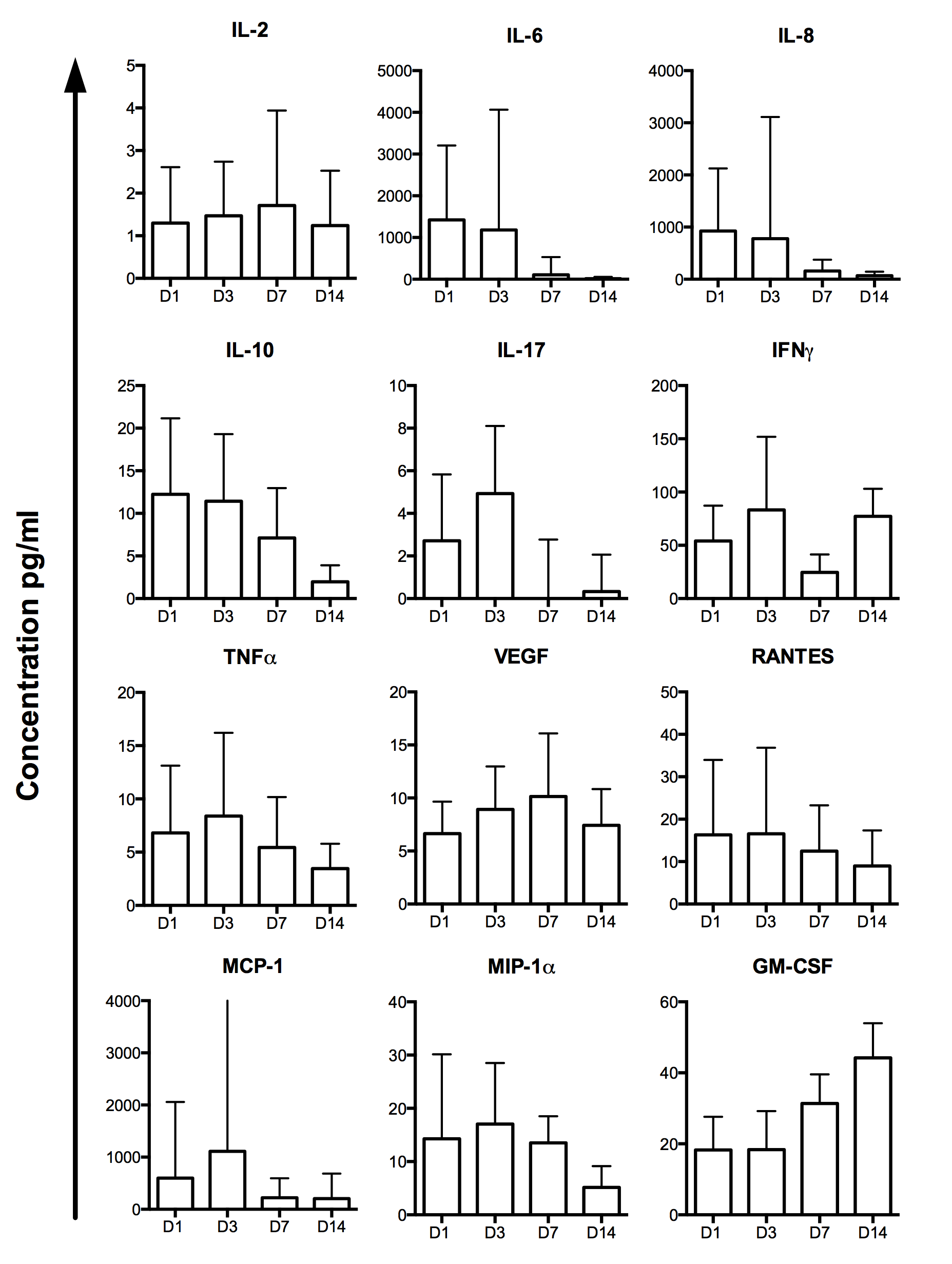

Supplement: S1 Fig — Median chemokine and cytokine levels, with error bars to 75th centile, are shown on days 1, 3, 7, and 14. No significant differences in the change in cytokine levels between days 1 and 3 were seen in the interferon-γ treated patients compared to controls. Between day 1 and day 7 there were larger reductions in IL-6, IL-17, RANTES and VEGF concentrations in IFNγ treated patients than controls, mirroring the more rapid decline in fungal burden (IL-6 5861pg/ml reduction versus 1500pg/ml, p = 0.03; IL-17 6.3pg/ml reduction versus 2.7pg/ml, p = 0.007; RANTES 16.7pg/ml versus 11.8pg/ml, p = 0.05; and VEGF 1.1pg/ml reduction versus 0.8pg/ml increase, p = 0.03). (TIFF) [file ppat.1004754.s001.tiff]

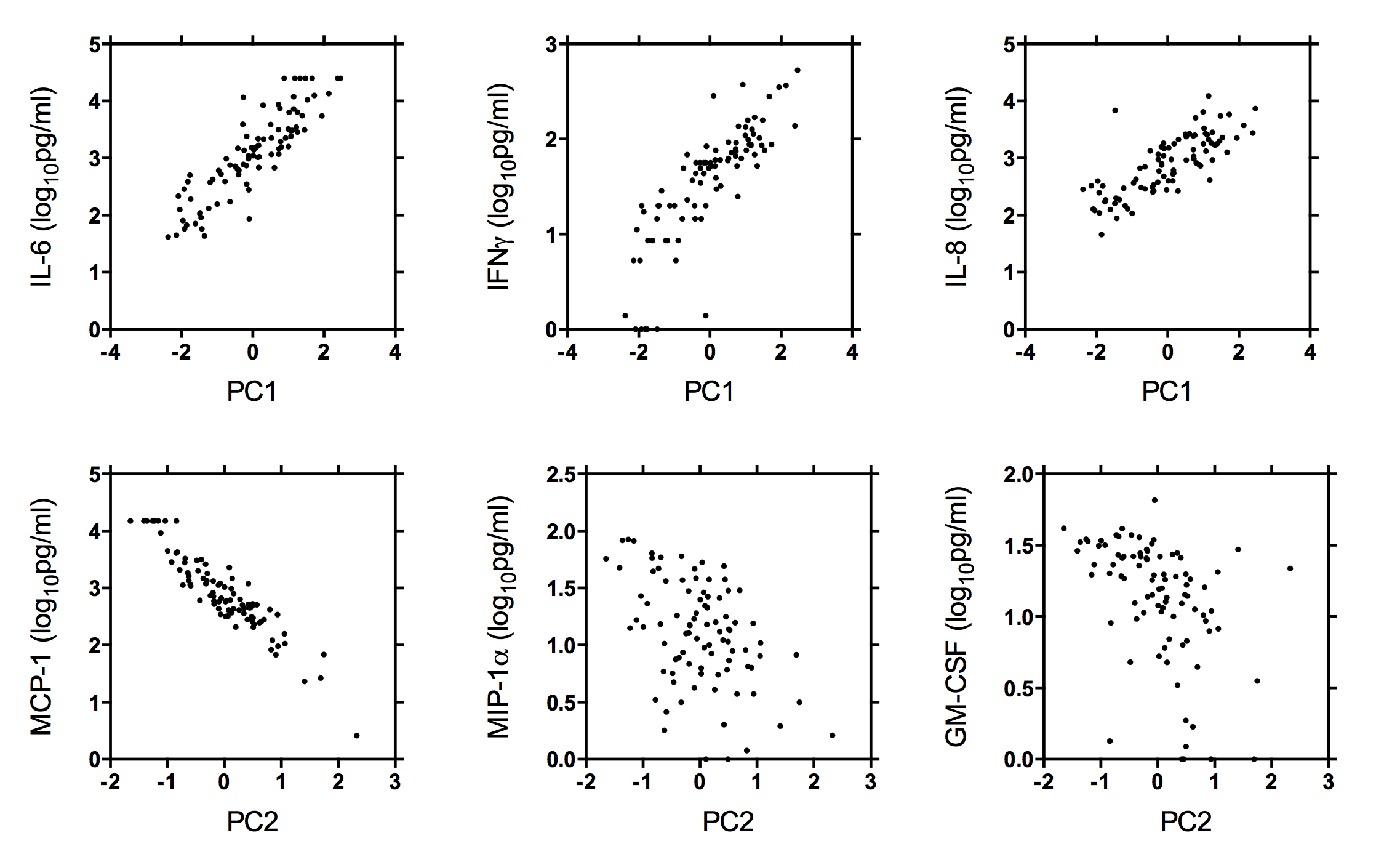

Supplement: S2 Fig — The associations between PC1 and baseline CSF IL-6, IFNγ, and IL-8 concentrations; and PC2 and baseline CSF MCP-1, MIP-1α, and GM-CSF concentrations. (TIFF) [file ppat.1004754.s002.tiff]
